# Sulphides from garlic essential oil dose-dependently change the distribution of glycerophospholipids and induce N6-tuberculosinyladenosine formation in mycobacterial cells

**DOI:** 10.1038/s41598-023-47750-0

**Published:** 2023-11-21

**Authors:** Rafał Sawicki, Jarosław Widelski, Wiesław Truszkiewicz, Sławomir Kawka, Guoyin Kai, Elwira Sieniawska

**Affiliations:** 1 Chair and Department of Biochemistry and Biotechnology, Medical University of Lublin, Chodzki 1, 20-093 Lublin, Poland; 2 Department of Pharmacognosy with Medicinal Plants Garden, Medical University of Lublin, Chodzki 1, 20-093 Lublin, Poland; 3 Medicofarma Biotech S.A., Zamenhofa 29, 20-453 Lublin, Poland; 4 School of Pharmaceutical Sciences, Academy of Chinese Medical Science, Zhejiang Chinese Medical University, Hangzhou, 310053 Zhejiang China; 5 Department of Natural Products Chemistry, Medical University of Lublin, Chodzki 1, 20-093 Lublin, Poland

**Keywords:** Biochemistry, Computational biology and bioinformatics, Drug discovery, Microbiology, Molecular biology

## Abstract

The antimicrobial properties of garlic are widely known, and numerous studies confirmed its ability to inhibit the growth of *Mycobacterium tuberculosis*. In this work, we explored the molecular mechanism of action of sulphides present in garlic essential oil against mycobacteria. The targeted transcriptomics and untargeted LC–MS metabolomics were applied to study dose- and time-dependent metabolic changes in bacterial cells under the influence of stressing agent. Expression profiles of genes coding stress-responsive sigma factors regulatory network and metabolic observations proved that sulphides from garlic essential oil are an efficient and specific agent affecting glycerophospholipids levels and their distribution within the cell envelope. Additionally, sulphides induced the Dimroth rearrangement of 1-Tuberculosinyladenosine to N6-tuberculosinyladenosine in mycobacterial cells as a possible neutralization mechanism protecting the cell from a basic nucleophilic environment. Sulphides affected cell envelope lipids and formation of N6-tuberculosinyladenosine in *M. tuberculosis.*

## Introduction

*Mycobacterium tuberculosis* is a Gram-positive bacillus causing highly infectious (especially in the initial phase) pulmonary tuberculosis (TB). Significant growth of the pathogen in the lungs is associated with extensive damage to the organism ultimately leading to death^1,2^. Moreover, TB and HIV are the main reason of mortality worldwide. According to the World Health Organization Global Tuberculosis Report, TB was the 13th leading cause of death worldwide and the top cause of a single infectious agent in 2019. In 2020 and 2021, it is anticipated that TB will rank as the second leading cause of death from a single infectious agent after COVID-19^3^. The COVID-19 pandemic has reversed years of progress in providing essential TB services and reducing the TB disease burden. Reduced access to TB diagnosis and treatment has increased in the TB incidence rate, which rose by 3.6% between 2020 and 2021, reversing declines of about 2% per year for most of the previous two decades^3^. Despite TB being a severe health, social and economic problem, tuberculosis therapy and searching for new drugs for TB treatment seems to be a neglected topic. Nowadays, therapy for TB is based on first-line drugs: isoniazid, ethambutol, rifampicin or streptomycin dispensed in the frame of the directly observed treatment short-course (DOTS)^4^. However, the drugs used to treat tuberculosis are fraught with many side effects, such as hepatotoxicity and nephrotoxicity, which is often the reason for discontinuing treatment. In addition, the occurrence of drug-resistant strains (multidrug- and extensively drug-resistant) reduces the effectiveness of the therapy^5^. Recently, there has been a paradigm shift in searching for potential new antimicrobials. The focus has moved to plants. Mainly due to the beneficial attributes that plants can confer over synthetic substances: high activity with a lack of adverse effects.

Garlic (*Allium sativum*, *Amaryllidaceae*) is the one of the oldest cultivated plant due to its nutritional properties, unique taste and aroma^6^. The application of garlic (*A. sativum*) in cooking, for the preservation of food, and generally in gastronomy has a tradition almost as old as human civilization^7,8^. According to an Egyptian medical papyrus (The Codex Ebers), dating to 1500 BC, garlic was a very popular spice, food ingredient, and medicine (for a variety of ailments: heart problems, headache, worms, and tumors) in Ancient Egypt^8,9^. Garlic was present during the first Olympic Games in Greece where vegetable has been consumed by athletes as a simulant^7,9^. Different preparations and extracts of garlic exert a bunch of different biological activities, including antibacterial, antiviral, antifungal and anthelminthic^6,10^. For centuries folk medicine used preparations based on garlic as the main ingredient for treating various types of infections, primarily the upper respiratory tract. Extracts of *A. sativum* have been demonstrated to inhibit the growth of susceptible, but more importantly, drug-resistant strains of *M. tuberculosis* at doses 1 and 3 mg/mL^11^. Generally, since the first report about the antimycobacterial activity of garlic, presented in 1946^12^, numerous studies have been carried out to demonstrate its preliminary in vitro potency against *M. tuberculosis*^13–15^. Most studies focused on isolated allicin or aqueous garlic extracts abundant in allicin and other thiosulfinates^5^. On the other hand, there are surprisingly few reports on the antimycobacterial effect of essential oil obtained from garlic bulbs (GEO). The phytochemical analysis of GEO showed that its composition is dominated by allyl polysulfides, including diallyl sulfide, diallyl disulfide, diallyl trisulfide, allyl methyl disulfide and allyl methyltrisulfide^16,17^. In studies by Swapna and co-workers, isolated allyl methyl trisulfide showed the highest inhibitory activity (125 µg/mL) against *M. tuberculosis*^5^. In another experiment, a garlic polysulfides mixture composed of 5% of diallyl monosulfide, 15% diallyl disulfide, 60% diallyl trisulfide, 20% diallyl tetrasulfide and less than 5% of diallyl pentasulfide and diallyl hexasulfide exhibited very strong antimycobacterial potential with the minimal inhibitory concentration of 2.5 µg/mL^6^. Nevertheless, the mechanism of action of GEO against *M. tuberculosis* was not explored so far.

In this work, we combined targeted transcriptomics and untargeted metabolomics to reveal dose- and time-dependent metabolic changes taking place in bacterial cells under the influence of GEO. Expression profiles of genes coding stress-responsive sigma factors regulatory network and metabolic observations proved that GEO is efficient and specific agent affecting glycerophospholipids levels and their distribution within the cell envelope. Additionally, sulphides induced the Dimroth rearrangement of 1-tuberculosinyladenosine (1-TbAd) to N6-tuberculosinyladenosine (N6-TbAd) in mycobacterial cells as a possible neutralization mechanism protecting the cell from a basic nucleophilic environment.

## Results

### GEO chemistry and activity

**Figure 1:**
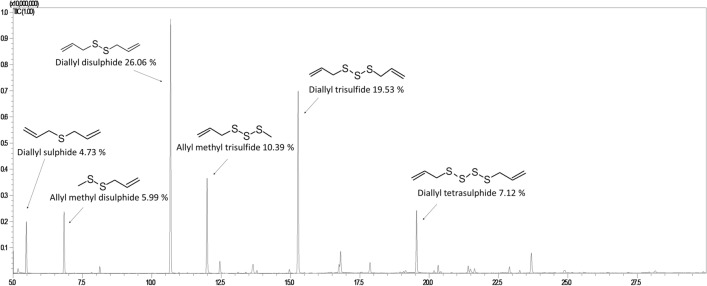
GC–MS chromatogram showing the chemical profile of garlic essential oil, and the percent contribution of its main constituents. The analysis was performed according to the following temperature programme: 50 °C held for 3 min, then was rised to 250 °C at a rate of 8 °C/min and this temperature was held for 2 min.

The chemical composition of purchased GEO was similar to that previously described in the literature sources. The main constituents were: diallyl sulphide 4.73%, allyl methyl disulphide 5.99%, diallyl disulphide 26.06%, allyl methyl trisulfide 10.39%, diallyl trisulfide 19.53% and diallyl tetrasulphide 7.12% (Fig. 1).

The MIC value of GEO against *M. tuberculosis* H37Ra was 0.032 g/L, while MIC of isoniazid and ethambutol were 0.00025 (1.82 µM) and 0.002 g/L (9.79 µM), respectively.

### Changes in the expression levels of genes coding sigma factors

**Figure 2:**
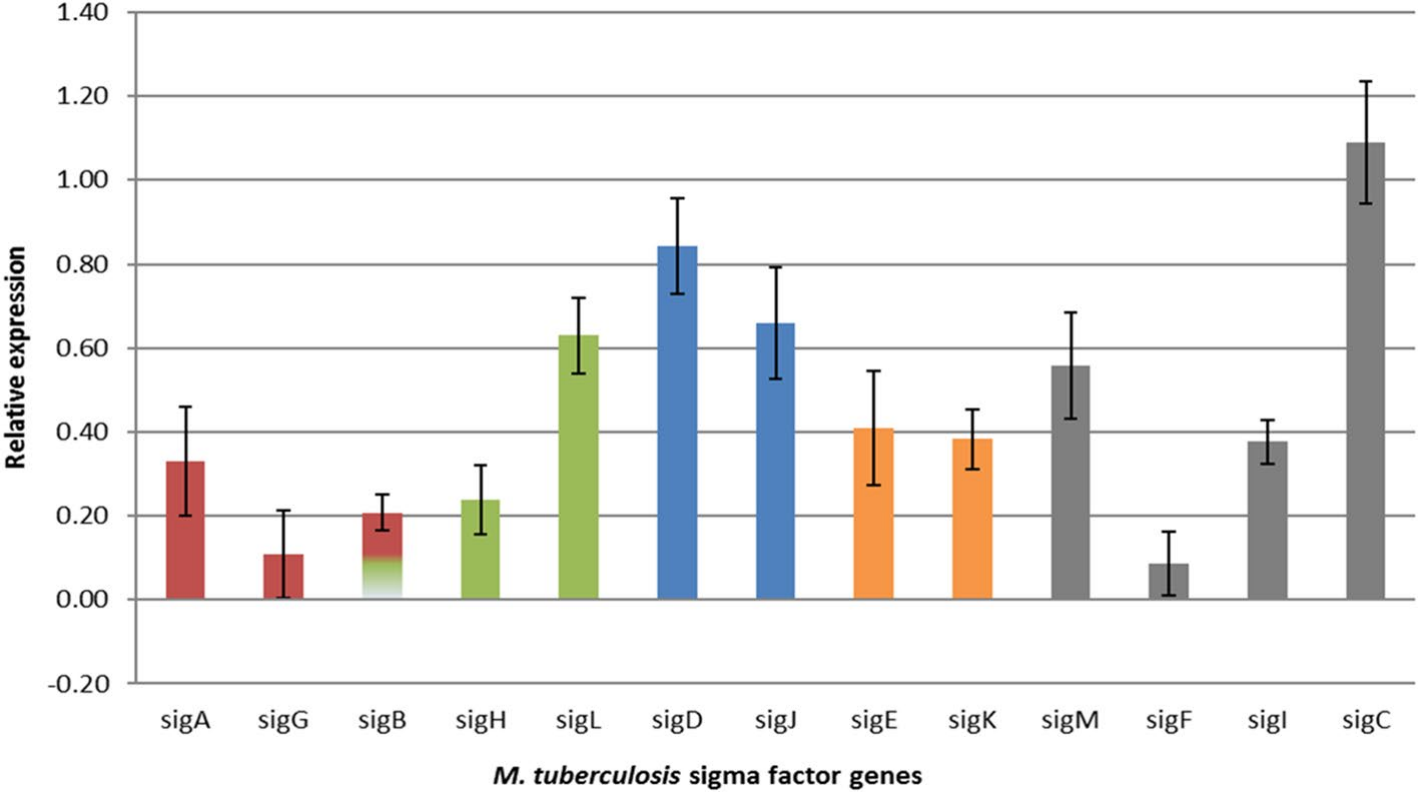
Relative fold change of *M. tuberculosis* sigma factor genes expression after 24 h exposition to higher dose of GEO. The colours represent five communities within the sigma factors regulatory network. The sigB gene and corresponding protein belong to two sigma factor communities. Distribution of sigma factors within families according to^19^. The value of 1 obtained for the control was subtracted the from all gene expression results.

The exposure of bacteria to GEO increased the expression of genes coding sigma factors. The most influenced was gene coding SigC, belonging to the largest core sigma factors community. Others highly upregulated were genes coding *sigJ* and *sigD*, which form a small family (Fig. 2).

### Lipids profile after bacteria exposure to lower dose of GEO

The pairwise analysis (treated bacteria vs control) showed no differences in lipophilic fraction after 24 h nor after 48 h (Figure S1A). The number of significantly changed lipids was small, and the fold change did not exceed 3 (Figure S2). Hence we concluded that a lower dose of GEO did not impact apolar lipids associated with bacterial cells.

**Figure 3:**
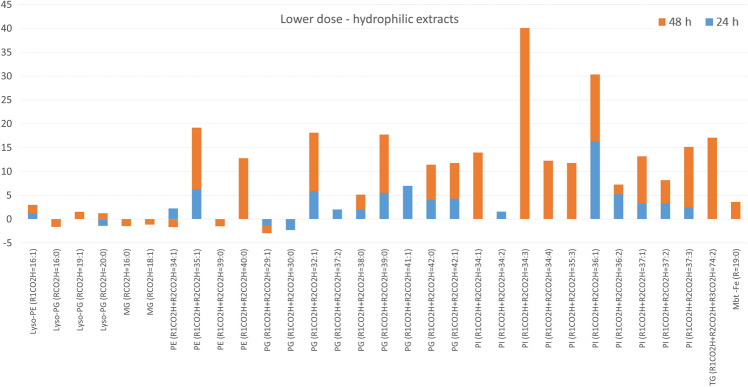
The fold change of lipids annotated in aqueous-methanolic extracts of bacterial pellets obtained after bacteria exposure to lower dose of GEO. Only lipids with *p* < 0.05 were included.

In methanolic-aqueous extracts the separation between groups was noticed after both incubation periods (Figure S1B). More than 30 lipids were assigned in each group, and the majority statistically significantly changed up to 16 fold in the first 24 h and up to 40 fold in the consecutive 24 h. Upregulation was predominant and included amphiphilic diacylglycerophosphoetanolamines (PE), diacylglycerophosphoglicerols (PG), and diacylglycerophosphoinositols (PI) (Fig. 3).

**Figure 4:**
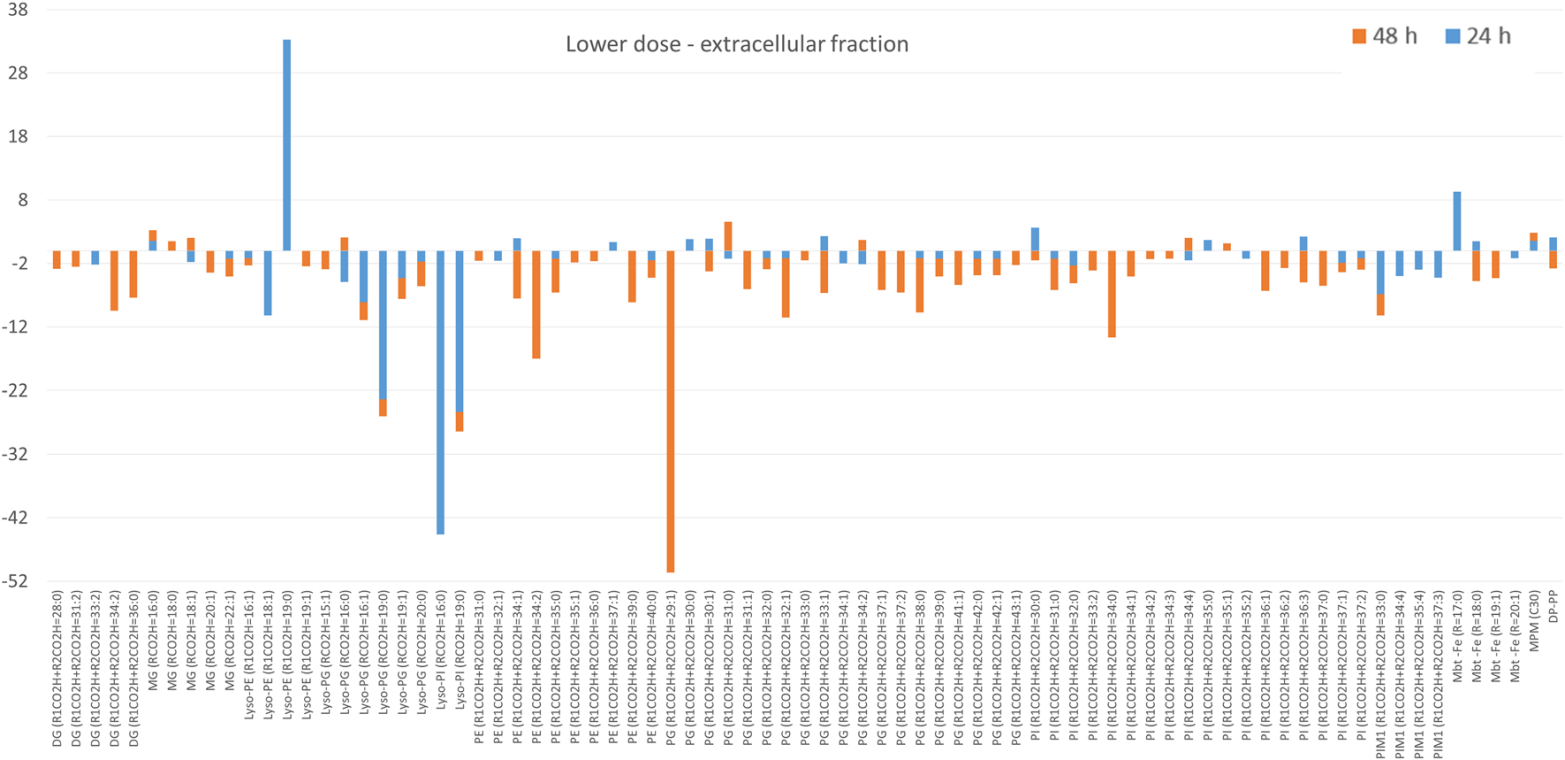
The fold change of lipids annotated in extracellular fraction obtained after bacteria exposure to lower dose of GEO. Only lipids with *p* < 0.05 were included. The graphs shows only features with fold change above 3.

The extracellular fraction of bacteria cultured with a lower dose of GEO differed from the control broth (Figure S1C). In this fraction, more than 70 lipids were assigned after both incubation times. Downregulation was significant, with Lyso forms of PE, PG, PI, and diacylglycerophosphoinositolmonomannosides (PIM1) affected after 24 h, and diacylglycerols (DG), PE, PG and PI affected after 48 h (Fig. 4).

### Lipids profile after bacteria exposure to a higher dose of GEO

**Figure 5:**
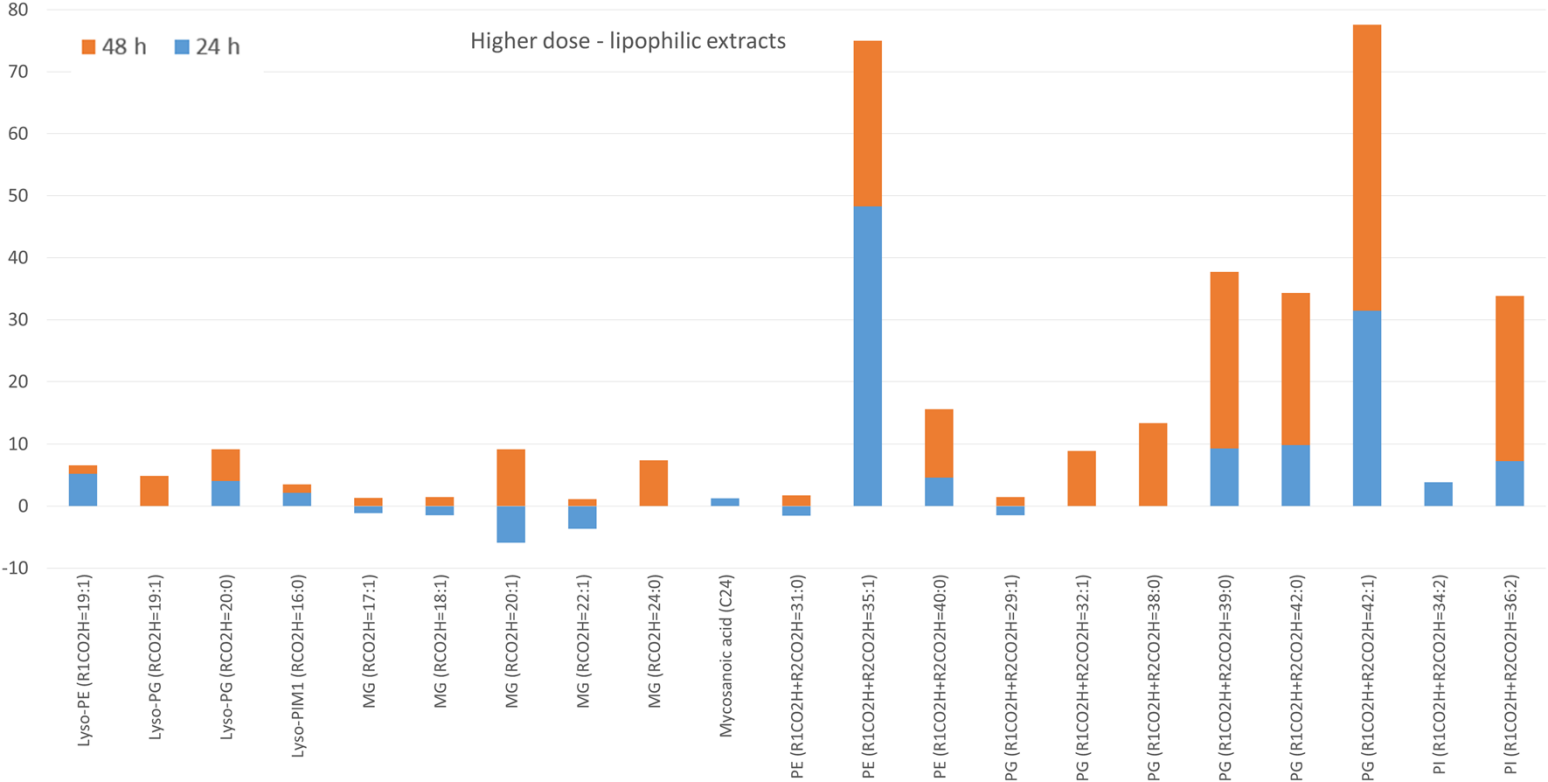
The fold change of lipids annotated in chloroform-methanolic extracts of bacterial pellets obtained after bacteria exposure to higher dose of GEO. Only lipids with *p* < 0.05 were included.

The lipophilic extracts prepared from the test culture and control bacteria differed significantly, with higher separation between groups after the second day of exposure (Figure S1D). Although small number of lipids was assigned in this fraction (less than 20 in each extract), their fold change was up to 48, and 46 after 24 and 48 h, respectively (Fig. 5). More pronounced upregulation of PE, PG, and PI was observed after 48 h.

The clear separation was observed in the pairwise analysis of methanolic-aqueous extracts obtained from the test and control group after incubation (Figure S1E). More than 50 compounds were assigned to each group. However only slight upregulation was noticed (up to 3.2 and up to 2.3 fold after 24 and 48, respectively) for all identified glycerophospholipids (Figure S3).

**Figure 6:**
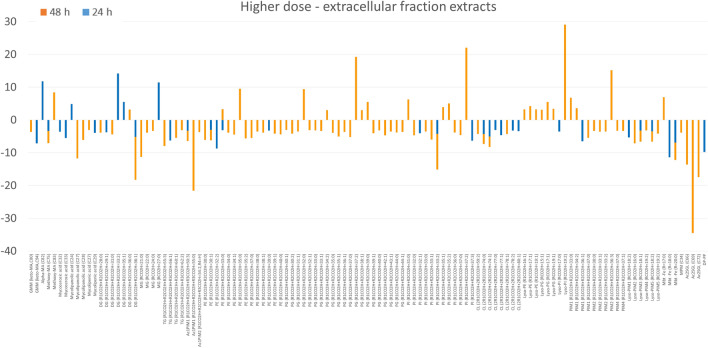
The fold change of lipids annotated in extracellular fraction obtained after bacteria exposure to higher dose of GEO. Only lipids with *p* < 0.05 were included. The graphs shows only features with fold change above 3.

In the extracellular fraction, test and control group were separated (Figure S1F). For GEO-exposed bacteria, the downregulation of lipids was noticed. Only several individual compounds and subclass of Lyso-forms of glycerophospholipids increased their levels after the second day of incubation (Fig. 6).

### TLC analysis of hydrolysed bacterial cells

**Figure 7:**
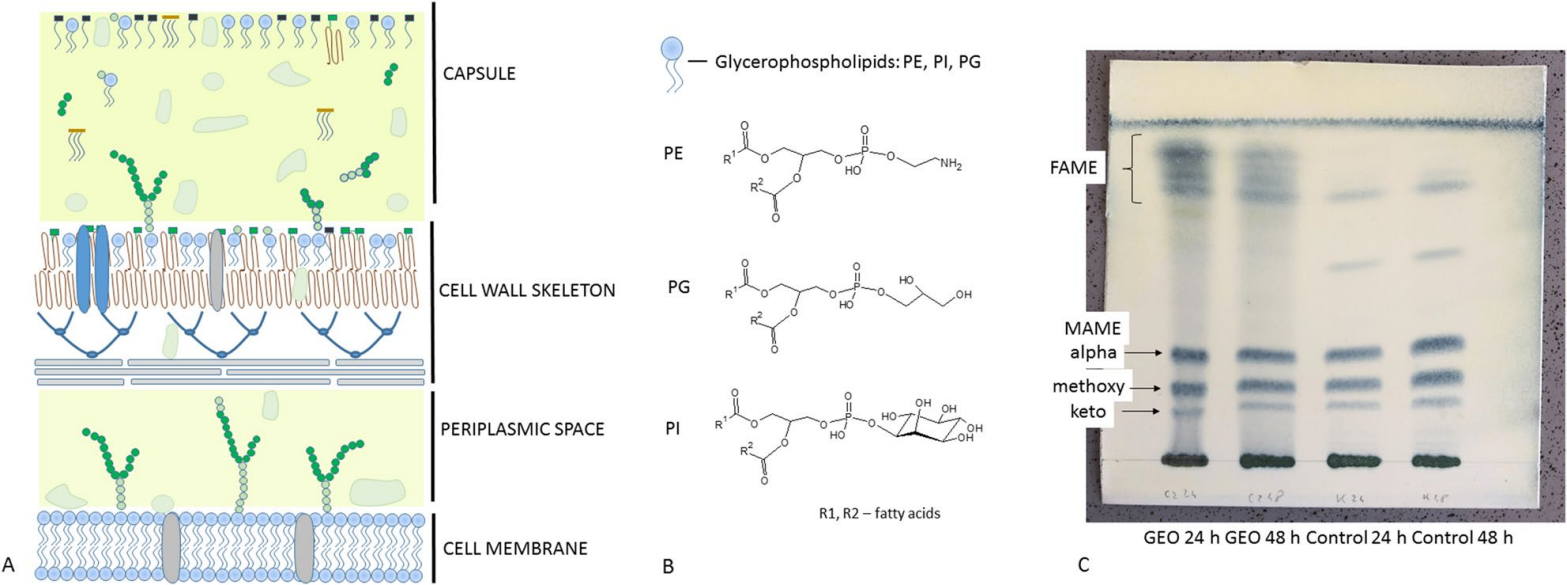
(**A**) Mycobacterial cell envelope and the position of glycerophospholipids within it; based on^28^; (**B**) Glycerophospholipids structures and the position of fatty acids in their molecules^29^. (**C**) TLC analysis of mycolic acids methyl esters and fatty acids methyl esters liberated though hydrolysis of bacteria exposed to higher dose of GEO and control bacteria. The compounds were visualised on TLC plate by spraying with 10% solution of phosphomolybdic acid and heating.

The hydrolysis of mycobacterial cells exposed to a higher dose of GEO liberated mycolic acids (MA) and fatty acids (FA), which after esterification were visualised as mycolic acids methyl esters (MAME) and fatty acids methyl esters (FAME). Test and control samples contained three subclasses of MAME: alpha, methoxy and keto, present in similar quantities. In the test sample, additional intense bands of FAME were observed after both incubation times (Fig. 7C).

### Identification of tuberculosinyladenosine isomers

**Figure 8:**
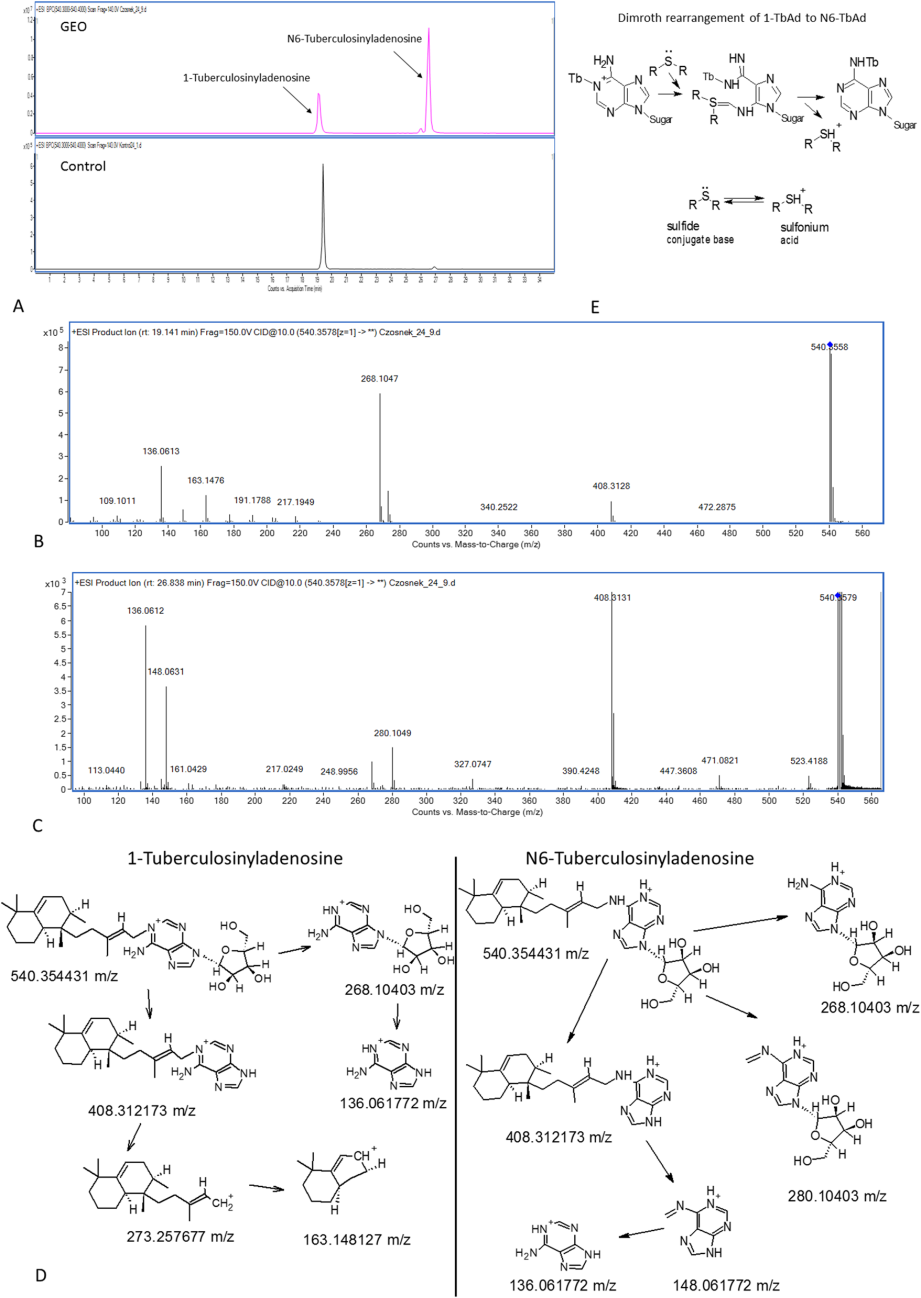
The tuberculosinyladenosine forms in lipophilic fraction of bacteria exposed to higher dose of GEO. (**A**) Chromatograms of test and control sample; (**B**) Mass spectrum of N6-tuberculosinyladenosine; (**C**) mass spectrum of 1-tuberculosinyladenosine; (**D**) fragmentation pathways of N6-tuberculosinyladenosine and 1-tuberculosinyladenosine; (**E**) the Dimroth rearrangement of 1-TbAd to N6-TbAd induced by sulphides present in GEO.

In the lipophilic extracts obtained in the experiment with a higher dose of GEO, two tuberculosinyladenosine (TbAd) isoforms were detected (Fig. 8A). Their mass spectra and proposed fragmentation pathways can be seen on Fig. 8B–D. 1-Tuberculosinyladenosine (1-TbAd) and N6-tuberculosinyladenosine (N6-TbAd) were differentiated based on the presence of the diagnostic ions resulting from the fragmentation of terpene core linked to adenosine moiety at first or sixth nitrogen atom. Fragmentation of ion at mz 408.3121, arising from 1-TbAd, gives daughter ions at mz: 273.2576 and 163.1481, while ion at mz 408.3121 in which adenosine is attached in N-6 position gives daughter ions at mz: 280.1040 and 148.0617 (Fig. 8D).

**Figure 9:**
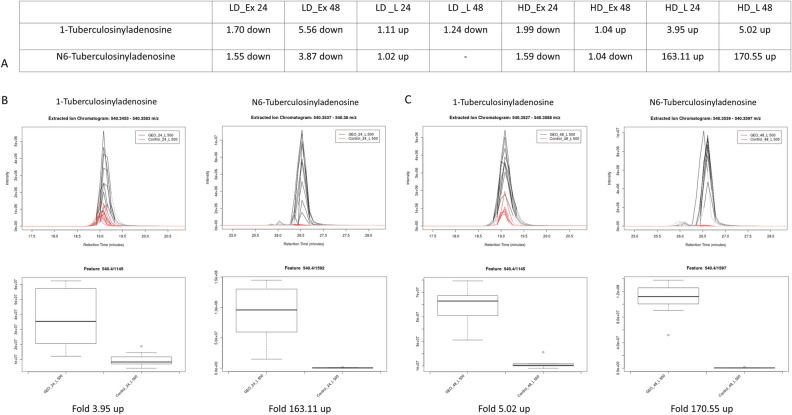
Tuberculosinyladenosine levels after the bacteria exposure to GEO. (**A**) The fold change of both isomers in different samples after 24 and 48 h of exposure; EIC chromatograms and box plots for 1-TbA and N6-TbA detected in lipophilic extracts obtained from bacterial pellet after first (**B**) and second (**C**) day of incubation. LD—low dose; HD—high dose; L—lipophilic extracts; Ex—extracellular fraction.

In the lipophilic samples obtained from cell pellets high and very high upregulation was observed for 1-TbAd and N6-TbAd, respectively (Fig. 9A–C). We also checked levels of both compounds in the samples obtained from extracellular fraction and after bacteria exposure to a lower dose of GEO (Fig. 9A). A higher and lower dose of GEO caused a decrease in TbAd levels in extracellular fraction. A lower dose of GEO increased levels of both isomers in the chloroform-methanolic extract. However, far less than it was observed for a higher dose.

## Discussion

*Mycobacterium tuberculosis* evolved a network of thirteen regulatory sigma factors responsible for programming of RNA polymerase to transcribe hundreds of specific genes and gene sets. The network controls the remodelling of metabolism in the adaptive bacterial response to environmental stimuli^18^. It contains five families (communities) working on three hierarchical levels (top level: SigA, SigB, SigH, SigM; middle level: SigE, SigG, SigL, SigJ, SigF, bottom level: SigD, SigK, SigI, SigC). Through the top sigma factors, the network is ready to provide a generic stress response before the stress-specific responses are expressed by lower levels of the network^19^. Environmental changes lead to the replacement of sigma factors in the RNA polymerase holoenzyme and, consequently, the transcriptional regulation of a different sets of genes. Competition among sigma factors for a limited amount of core RNA polymerase makes a basal regulatory mechanism for managing mycobacterial gene expression in response to diverse environmental signals^20,21^. Thus, sigma factors support the production of specific protein sets, which help the bacteria survive under stress conditions^22^. However, most of the sigma factors demonstrate the extra-cytoplasmic function (ECF). That means that sigma factors proteins are blocked by pairing with corresponding antisigma factors. Four create antisigma factor, membrane-anchored pairs: SigL/RslA, SigK/RskA, SigM/RsmA, and SigD/RsdA and in such form, await extracellular stimuli. Once activated, sigma factors control, for example, cell envelope synthesis, secretory functions, periplasmic protein repair and degradation^18^.

We profiled the whole set of thirteen sigma factors transcription patterns. Since sigma factors regulate numerous genes and processes, we hoped for more specific suggestions concerning GEO possible mode of action. All the genes were slightly overexpressed, suggesting that GEO treatment leads to global metabolic adaptations. In that case, it seems to be primarily a mechanism to respond to the drug action. On the other side, the observed transcripts ratios were only static insight into the state of the expression pattern of sigma genes after a day-long exposure to GEO. So we could miss more dynamic transcriptional shifts earlier during drug exposition. Nevertheless, 24 h of bacteria exposure to GEO resulted in the highest upregulation of genes coding two bottom-level sigma factors: *sigD* and *sigC*. Genes coding middle-level factors *sigL* and *sigJ* were less affected, suggesting that bacteria triggered stress-specific responses. Little is known about the exact regulon of SigC, but SigD exhibits ECS function and regulates the fatty acid metabolic process^23^. Since the transcripts of *sigD* were still needed after 24 h, our observations indicate GEO's long-lasting and stress-specific influence on bacteria. Also, to unblock SigD from its antisigma factor, the stress factor must reach the mycobacterial cell membrane.

Experiments accomplished in this study enabled the dose-dependent observations of lipid profiles of mycobacteria under the influence of GEO. The mycobacterial cell wall can be divided into three main compartments: cell membrane, cell wall skeleton (comprised of peptidoglycan-arabinogalactan-mycolate) and capsule (formed from lipids placed in the polysaccharide-protein matrix). Lipids from the outermost layer of cell envelope are non-covalently attached and can be released into the culture medium^24^ (Fig. 7A). Bacteria exposure to lower dose of GEO did not affect apolar lipids associated with the cell wall (lipids extractable with chloroform–methanol), but upregulated lipids involved in the formation of the cell membrane (amphiphilic glycerophospholipids extractable with mixture of methanol and water). Numerous PE, PI and PG were detected in increased amounts, especially after the second day of exposure. The mycobacterial plasma membrane constitutes cardiolipin (CL), PE, PI, and phosphatidylinositol mannosides (PIM). Diacylglycerophosphoglicerol is less abundant in mycobacteria however, required for CL biosynthesis. The production of one CL moiety utilizes two PG molecules^25^. Diacylglycerophosphoinositol is constantly turned over within 7 h, what suggests the continuous renewal of PI pool within bacterial cells. Due to this fact bacteria are always ready to use PI for the biosynthesis of PI mannosides (PIM) or to degrade it to lyso forms^26^. It can be hypothesized that similar turnover can occur in PE and PG cases. The increase of cell membrane constituents was prolonged and even more pronounced after 48 h of bacteria incubation with a lower dose of GEO. It confirms higher metabolic activity related to formation of cell membrane building blocks. However, glycerophospholipids are also present in the cell wall skeleton and in a capsule. Bacteria exposed to a lower dose of GEO responded with the downregulation of glycerolipids and glycerophospholipids, which are released to the growth medium in normal conditions (control culture medium). After the first day of exposure, lower levels of Lyso-PE, Lyso-PG, Lyso-PI (monoacylated forms), and PIM1 were detected. The subsequent incubation day significantly decreased the amounts of PE, PG, and PI in the medium. This observation may indicate the inhibited transfer of these molecules to the outermost part of the cell envelope. In normal conditions, phospholipids are present in the capsule as noncovalently attached molecules^27,28^; however, the influence of a lower dose of GEO disturbed the distribution of these lipids.

The bacteria exposure to a higher dose of GEO reversed the trend of lipids distribution between chloroform-methanolic and aqueous-methanolic extracts. The high fold upregulation was observed for PE, PG and PI in chloroform-methanolic extract, suggesting that a portion of these molecules was enriched in the cell wall. A significant number of lipids annotated in the aqueous-methanolic extract was only slightly upregulated, indicating a visible but minor influence of GEO at the deeper layer of the cell envelope. The profile of extracellular fraction obtained after bacteria exposure to a higher dose of GEO was similar to that after exposure to the lower dose, with predominant downregulation.

Our observations indicate that the GEO dose independently inhibited standard release of lipid molecules into the culture medium. Bacteria sealed up cells in response to the stressing agent. Interestingly GEO dose dependently changed the distribution of PE, PI and PG within bacterial cell envelope. After bacteria treatment with a higher dose, PE, PI and PG molecules were extracted in the first step (chloroform-methanolic extract), meaning that they were more readily available, most probably placed within the more external compartment of the cell envelope. The increase in PE, PI and PG levels correlated with abundant amounts of fatty acids in test bacterial cell hydrolysates, as was revealed by TLC analysis (Fig. 4C). Diacylglycerophosphoethanolamines, PI and PG lipids are acylated with two fatty acids. molecules at the glycerol moiety^29^ (Fig. 7B), Hence the upregulated formation of these structures explains the induced production of fatty acids, which were incorporated into glycerophospholipids. The TLC analysis also indicated that GEO did not influence levels of mycolic acids, suggesting that the mycobacterial mycomembrane (mycolic acids layer) was not affected in the experiment.

Tuberculosinyladenosine isomers are modified adenosine derivatives produced only by pathogenic mycobacterial strains^30^. 1-TbAd was proposed as a marker of Mtb infection and TB disease because it was detected in clinical isolates. N6-TbAd variant was also described in vivo during infection in mice^31^. Bacteria exposed to GEO responded with decreased levels of both TbAd isomers in the culture medium In contrast a higher dose of GEO caused a significant upregulation of 1-TbAd and N6-TbAd in the extract obtained from the bacterial pellet. This observation suggests that the site of action of GEO constituents is the cell envelope or cytosolic compartment. Stressed bacteria considerably increased (24 h) and maintained increased (48 h) cellular levels of N6-TbAd (170 fold up in comparison to control). N6-TbAd is not a primary product of diterpene synthase (Rv3378c), indicating that in bacteria, it arises from 1-TbAd^32^. The biological origin of N6-TbAd is the non-enzymatic conversion of 1-TbAd to N6-TbAd in an isomerization reaction called Dimroth rearrangement. This chemical process requires an attack of a nucleophile at C-2 to open the ring, which then closes at the unsubstituted nitrogen to give the N6-linked form^32^. N6-TbAd isomer can be produced in different cellular compartments; however, Dimroth rearrangement is favoured under basic conditions^32^. In an acidic environment, the concentration of nucleophiles is low. Thus 1-TbAd is less prone to rearrangement^32^ and plays the role of proton scavenger^31^. The main GEO constituents are: diallyl sulphide 4.73%, allyl methyl disulphide 5.99%, diallyl disulphide 26.06%, allyl methyl trisulfide 10.39%, diallyl trisulfide 19.53% and diallyl tetrasulphide 7.12% Sulfides or thioethers (R − S − R') are organosulfur functional groups containing one or more S2^−^ ions. Chemically, sulfides are strong bases with nucleophilicity much greater than oxygen^33^. Sulphides induced the Dimroth rearrangement of 1-TbAd to N6-TbAd in mycobacterial cells. A proposed reaction (Fig. 8E) shows that sulphides can be transformed to sulfonium ions as a possible neutralization mechanism and that conversion of 1-TbAd to N6-TbAd protects the cell from a basic nucleophilic environment. So far, it has been known that 1-TbAd accepts protons in an acidic environment within macrophages and modulates Mtb’s intracellular acidic growth niche within a host^31^. N6-TbAd was proposed as an altered, possibly inactivated form of 1-TbAd^32^. However, our observations suggest that the rearrangement of 1-TbAd to N6-TbAd can be an essential mechanism compensating the influence of a basic environment on mycobacteria. Ghanem and co-workers indicated that 1-TbAd could pass the cytosolic membrane into the periplasm, mycolate membrane, or surface targeting, or protecting specific molecules during exposure to low pH in vitro, without altering intracellular pH of bacteria^34^. The similar shift of 1-TbAd to the basic compartments is questionable because 1-TbAd passes membranes in an uncharged form. In contrast, as suggested by Young and co-workers (2015)^32^, the Dimroth rearrangement undergoes 1-TbAd + form, which is membrane-impermeable. Predominantly charged at neutral pH 1-TbAd + ^31^ is ready to interact with a basic molecules penetrating cellular compartments. This is in agreement with the existing suggestion that undissociated sulfides permeate through bacterial membranes^35^ and preferentially act on the hydrocarbon cores rather than the hydrophilic surfaces of lipid bilayers^36^. The increased production of cell membrane phospholipids observed after mycobacteria exposure to GEO proved that these molecules are the sites of action of garlic sulphides. The higher dose of stressing agent induced the redistribution of PE, PI and PG within the bacterial cell envelope, possibly moving them to a more external compartment of the cell envelope as a trap, preventing the penetration of sulphides to deeper compartments of the cell.

## Materials and methods

### Characterization of the garlic essential oil

The commercially available GEO (Nanga, Poland) was used in experiments. Its chemical composition was checked employing gas chromatography–mass spectrometry according to the methodology described previously^37^. The analysis was performed on Shimadzu GC-2010 Plus coupled to a Shimadzu QP2010 Ultra mass spectrometer (Shimadzu, Japan) with a fused-silica 30 m, 0.25mm i.d., 0.25mm film thickness capillary column ZB-5 MS (Phenomenex, USA). Homologous series of n-alkanes (C8-C24) was used to determine retention indices under the same operating conditions. Identification was performed by comparison of mass spectra of compounds and their retention indices with computer-supported spectral library (NIST database) and literature data.

### Bacteria and culture conditions

*M. tuberculosis* H37Ra (ATCC25177) was grown on Löwenstein-Jensen slopes (BioMaxima, Lublin, Poland) for two weeks, then transferred to fresh Middlebrook 7H9 broth supplemented with 10% ADC and 0.2% glycerol (MilliporeSigma, St Louis, USA). The preparation of inoculum and the determination of minimum inhibitory concentration (MIC) were described in detail in our previous work^38^.

High-density cultures used in metabolomic experiments (around 1 × 10^9^ CFU/mL; 400 mL) were obtained from freshly prepared inoculum (4 mL each), which was propagated for four weeks at 37°C with aeration (100 rpm). After that time, the test cultures were supplemented with lower (0.512 mg/mL) and higher (4 mg/mL) dose of GEO dissolved in DMSO, while 2% of DMSO was added to the control cultures. To determine the effective doses in the high-density cultures the serial two-fold dilutions of GEO were placed in 96 wells plate and supplemented with 50 μL of *M. tuberculosis* inoculum (around 1 × 10^9^ CFU/mL) and 15 μL of Alamar Blue dye. The plate was sealed and incubated overnight at 37 °C. The change in colour of the Alamar blue from blue to pink was assessed. The effective doses were confirmed not to influence the bacteria metabolic activity because the wells supplemented with GEO in the concentration of 0.512 mg/ml and 4 mg/ml were pink.

Cultures were incubated for 24 h and 48 h in the same conditions. The bacterial metabolism was stopped by adding cold methanol (-60°C) (1:1v/v). Cultures were aliquoted in 50 mL Falcon tubes and centrifuged for 30 min at 8000 rpm at 4°C to harvest bacteria. The supernatant was removed and collected, while pellets were rinsed three times with cold phosphate-buffered saline (Biomed, Lublin, Poland) and centrifuged again to remove traces of medium. The obtained bacterial biomass was lyophilized and stored at -60°C until extracted.

### Bacterial metabolites extraction and analysis

For the extraction of extracellular lipophilic compounds (Ex) culture supernatants were pooled and concentrated under reduced pressure at 37 °C. The obtained 50 mL of each culture was extracted thrice with 100 mL of the mixture of chloroform and methanol (2:1 v/v). The organic phase was aliquoted in 30 mL (7 technical replicates) and evaporated to dryness, leaving a fatty residue. The samples were stored at – 20 °C before analysis.

For the extraction of cellular metabolites, the lyophilized bacterial biomass was weighted (30 mg per sample, 6–10 technical replicates), poured with the mixture of chloroform, methanol and water (2:1:0.1; v/v/v; 1.5 mL) for lipids extraction (L), and sonicated 20 min. After centrifugation (10 min at 13,000 rpm at 4 °C) the supernatant was collected, while bacterial residue was extracted again. Combined supernatants were evaporated to dryness under reduced pressure at 30 °C. The bacterial pellets were poured with a mixture of methanol and water (1:1; v/v; 1.5 mL) for extraction of more polar compounds (H), and the procedure was performed as described above. The extracts were stored at -20 °C before analysis.

Untargeted liquid chromatography—mass spectrometry analysis of bacterial metabolites was done on Agilent 1200 Infinity HPLC chromatograph coupled to Agilent 6530B QTOF spectrometer equipped with Dual Agilent Jet Stream spray source (ESI) (Agilent Technologies, Santa Clara, CA, USA. Extracted metabolites were dissolved in acetonitrile–methanol-isopropanol (1:1:2 v/v/v), filtered through 0.22 µm PTFE syringe filters and separated on Gemini® chromatographic column (3 µm i.d. C18 with TMS endcapping, 110 Å, 150 × 2 mm) supported by guard column (Phenomenex Inc, Torrance, CA, USA). Chromatographic and mass spectrometry conditions were the same as previously published^38^. The acquisition was performed in a positive ion mode taking two spectra/s in a scan range from 100 to 3000 m/z.

Raw data obtained from LC–MS analysis were explored and converted to mzDATA format in Mass Hunter Qualitative Analysis software (version B.07.00; Agilent Technologies, Santa Clara, CA, USA). For the feature extraction, an open–source software XCMS (version Version 3.7.1) was used^39^. The Obiwarp method was applied for the correction of retention time, followed by the normalization, scaling and filtering done before pairwise statistical analysis. The calculations were based on the intensities of all aligned features. The intensity threshold was set at 500; features were retained only if present in at least 5 replicates and considered statistically significant at p < 0.05 by unequal variances Welch t-test. Principal component analysis plot (PCA) was performed on the centered and scaled data. MS-LAMP software (http://ms-lamp.igib.res.in) was used to annotate mycobacterial lipids, while other compounds were tentatively identified based on their fragmentation patterns and PubChem database (https://pubchem.ncbi.nlm.nih.gov).

### Thin layer chromatography (TLC) analysis of hydrolysed bacterial cells

The procedure of hydrolysis and methylation of mycolic acids was adopted from^40^ and performed with some modifications. 100 mg of lyophilized bacterial biomass obtained in the experiment with a higher dose of GEO was weighed into a glass tube and suspended in 2 mL of water and 2 mL of 40% tetrabutylammonium hydroxide. The suspension was heated at 100 °C for 20 h. After cooling the suspension, 200 μL of iodomethane and 4 mL of dichloromethane were added. The mixture was shaken for one hour at room temperature, after which the organic phase was washed with 2 mL of 1 M HCL and then with 2 mL of water. The organic phase was evaporated, and the residue was dissolved in 400 μL of dichloromethane. Equal amounts of the resulting mixtures (mycolic acids methyl esters, MAME and co-extracted and co-methylated shorter-chain fatty acids, FAME from control and test samples) were applied to a silica gel plate. The plate was developed three times with a mixture of hexane and ethyl acetate (95:5 v/v). After drying, sprayed with a 10% solution of phosphomolybdic acid and heated until spots appeared.

### Gene expression analysis

The test culture supplemented with a higher dose of GEO and the corresponding control culture were used for gene expression analysis. After 24 h incubation and before the metabolite quenching, 2 mL of each culture was collected and centrifuged. Total RNA extraction (FastRNA Pro Blue Kit; MP Biomedicals, Santa Ana, USAMPBiomaterials) and qPCR reactions (LightCycler®EvoScript RNA SYBR®Green I Master kit; Roche, Basel, Switzerland) were performed according to manufacturer’s instructions and described with details in our previous work^38^. The primer sequences can be found in Table S1. For the relative quantification of transcripts, targets were normalized to 16S rRNA, and relative mRNA quantification was calculated according to the delta delta mathematical model.

## Conclusion

Our observations indicate that the GEO dose- independently inhibited the release of lipid molecules into the culture medium, while dose- dependently changed the distribution of PE, PI and PG within bacterial cell envelope. Lower dose increased the amounts of phospholipids at the cell membrane level. In contrast, higher dose induced the shift of these molecules to the more external compartment of the cell envelope without impacting mycolic acids. GEO increased the number transcripts of stress responsive sigma factors and induced the Dimroth rearrangement of 1-tuberculosinyladenosine to N6-tuberculosinyladenosine in mycobacterial cells.

### Supplementary Information

Supplementary Information.

## Data Availability

The datasets analysed during the current study are available at https://doi.org/10.5281/zenodo.10134365.
