# Supplementary material for: Sulphides from garlic essential oil dose-dependently change the distribution of glycerophospholipids and induce N6-tuberculosinyladenosine formation in mycobacterial cells
Source: Sci Rep. 2023 Nov 21;13:20351. doi: 10.1038/s41598-023-47750-0 (PMC10663513; doi:10.1038/s41598-023-47750-0)
Supplement: Supplementary file 1 — Supplementary Information. [file 41598_2023_47750_MOESM1_ESM.pdf]

Sulphides from garlic essential oil dose-dependently change the distribution of glycerophospholipids and induce N6-tuberculosinyladenosine formation in mycobacterial cells

Rafał Sawicki[1], Jarosław Widelski[2], Wiesław Truszkiewicz[2], Sławomir Kawka[3], Guoyin Kai[4], Elwira Sieniawska[5*]

[1] Chair and Department of Biochemistry and Biotechnology, Medical University of Lublin, Chodzki 1, 20-093 Lublin, Poland; rafal.sawicki@umlub.pl; wieslaw.truszkiewicz@umlub.pl

[2] Department of Pharmacognosy with Medicinal Plants Garden, Medical University of Lublin, Chodzki 1, 20-093 Lublin, Poland; jwidelski@pharmacognosy.org

[3] Medicofarma Biotech S.A. Zamenhofa 29, 20-453 Lublin, Poland; slawomirkawka@o2.pl

[4] School of Pharmaceutical Sciences, Academy of Chinese Medical Science, Zhejiang Chinese Medical University, Hangzhou, Zhejiang, 310053, China; guoyinkai1@126.com

[5] Department of Natural Products Chemistry, Medical University of Lublin, Chodzki 1, 20-093 Lublin, Poland

*Correspondence: esieniawska@pharmacognosy.org

Table S1. Primers used for qPCR analyses

Gene	Primer pair (5′–3′)
sigA:	FOR: GACGAAGACCACGAAGAC
	REV: TCATCCCAGACGAAATCAC
sigB:	FOR: CTCGTGCGCGTCTATCTGAA
	REV:AGCAGATGCTCGGCATACAA
sigE:	FOR: AACCCCGAGCAGATCTACCA
	REV: CTCGATGTCACACAGCACCA
sigG:	FOR: CGTCAATGAGCCTACGCAGA
	REV:GCGAAATTCCGTTCAGTCCG
sig H:	FOR: GCCGCTGTTTCTTGCGATAG
	REV: CCAGGAGACGATGGTGAAGG
sig M:	FOR:CGTCAGCAGTTGGTTGCAC
	REV: ACATCTTCTAGAGGGGCGGT
sig L:	FOR: CGTGATCCAGCGGTCCTAC
	REV:CAATCGCGACTTCACCGTTC
sig J:	FOR:GACCAGCCCGAGTATGAACC
	REV:ATCCCGACGTGACGTTTACC
sig F:	FOR:CCGCAGATGCAGTTCCTTGA
	REV: GGTCGGACTTCGTCTCCTTC
sig D:	FOR: AACAATCTCGTCCTTCAGCCG
	REV:CGAGATCCTCATTCTGCGTGT
sig K:	FOR: CCGCCACACCTCAAGATAGA
	REV: TCTACGACCACACCAAGTCG
sig C:	FOR: TTACCGCACTCGCCTTGTC
	REV: GGACAGATAGGCGACGAACC
sig I:	FOR:AAGACATGGTGCAAGAGGCA

	REV: ACGCCGACTTGATGTGATCC
16S RNA	FOR: ACTTCGGGATAAGCCTGGGA
	REV: AGCGCTTTCCACCACAAGAC

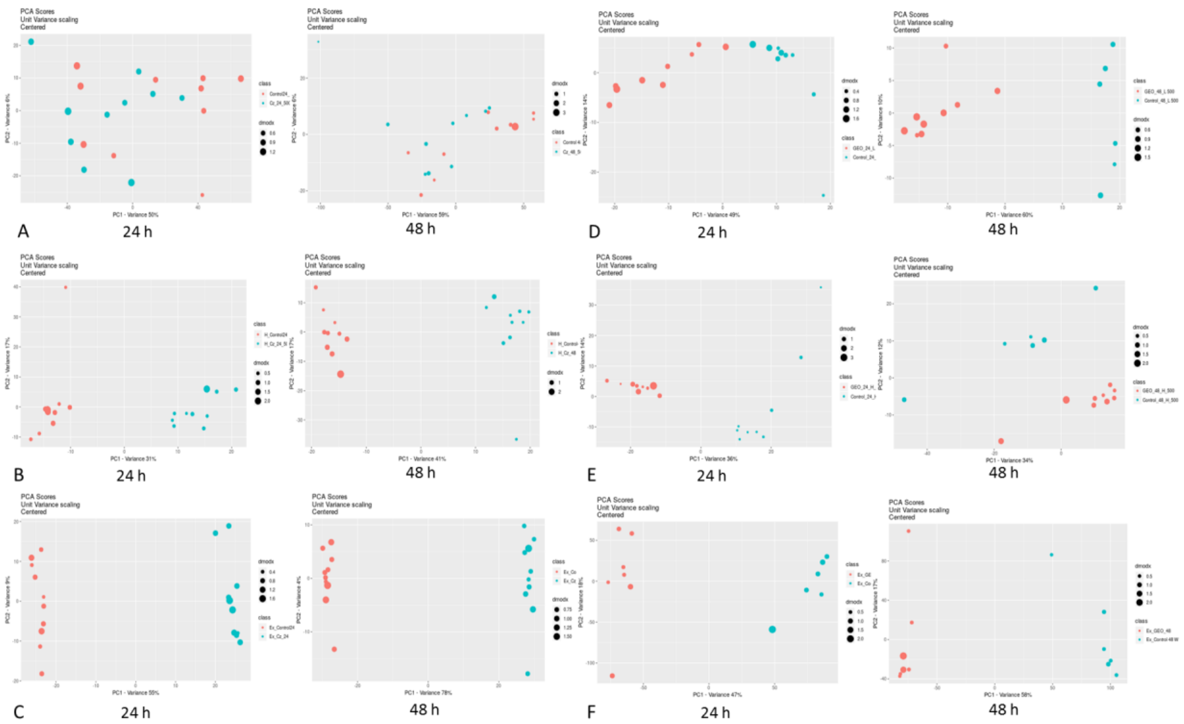

Figure S1. Figure 3. Principal component analysis scores from pairwise analysis (XC-MS online) showing the separation between test and control groups. A – lower dose experiment (LD) lipophilic extracts; B – LD hydrophilic extracts; C – LD extracellular fraction; D – higher dose experiment (HD) lipophilic extracts; E – HD hydrophilic extracts; F – HD extracellular fraction; DmodX: The distance between the original data point and the model plane in X-space.

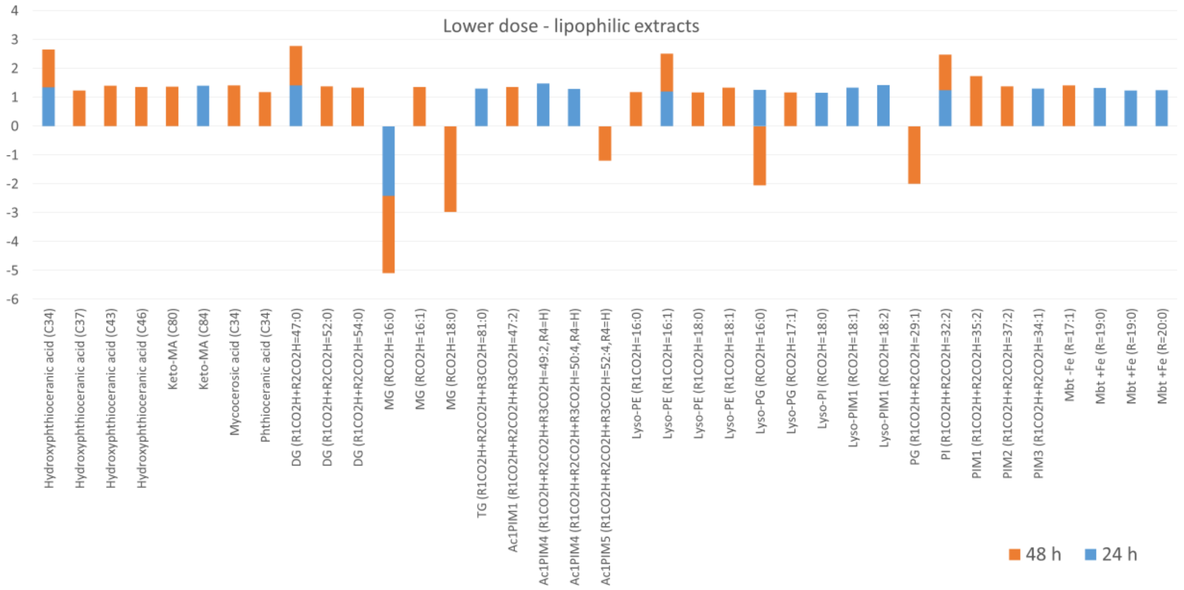

Figure S2. The fold change of lipids annotated in chloroform-methanolic extracts of bacterial pellets obtained after bacteria exposure to lower dose of GEO. Only lipids with $p<0.05$ were included.

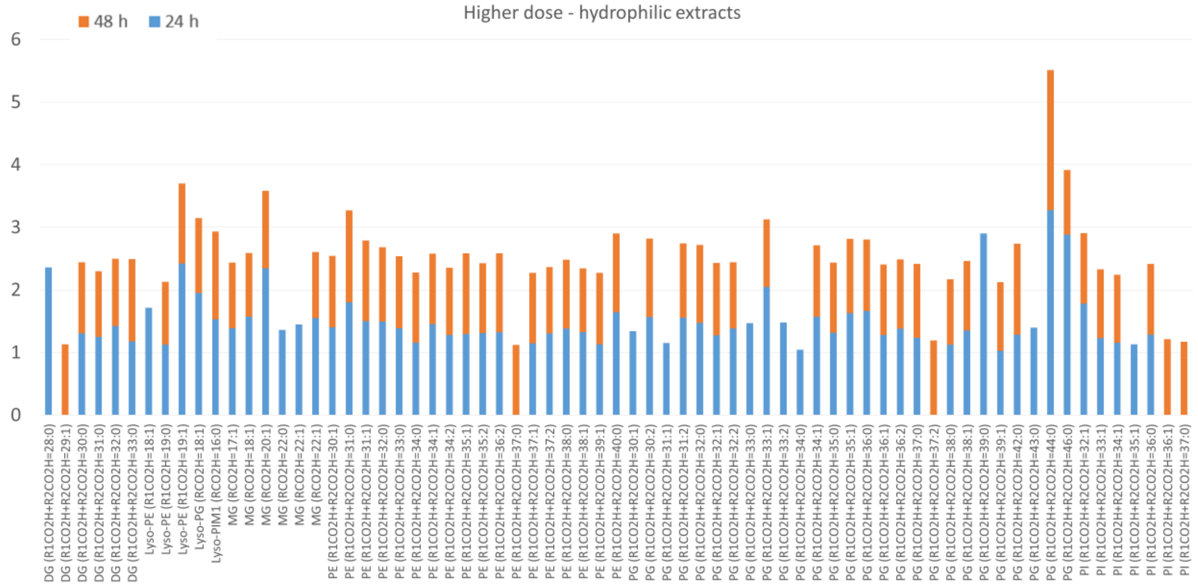

Figure S3. The fold change of lipids annotated in aqueous-methanolic extracts of bacterial pellets obtained after bacteria exposure to higher dose of GEO. Only lipids with $p<0.05$ were included.
